# Portable Point-of-Care Device for Dual Detection of Glucose-6-Phosphate Dehydrogenase Deficiency and Hemoglobin in Low-Resource Settings

**DOI:** 10.3390/bios15090577

**Published:** 2025-09-03

**Authors:** Rehab Osman Taha, Napaporn Youngvises, Runtikan Pochairach, Papichaya Phompradit, Kesara Na-Bangchang

**Affiliations:** 1 Graduate Program in Bioclinical Sciences, Chulabhorn International College of Medicine, Thammasat University, Pathum Thani 12120, Thailand; rehab.osm@dome.tu.ac.th (R.O.T.); papichph@tu.ac.th (P.P.); 2 Drug Discovery and Development Center, Office of Advanced Science and Technology, Thammasat University, Pathum Thani 12120, Thailand; napaporn@tu.ac.th (N.Y.); runtikan@tu.ac.th (R.P.); 3 Center of Excellence in Pharmacology and Molecular Biology of Malaria and Cholangiocarcinoma, Chulabhorn International College of Medicine, Thammasat University, Pathum Thani 12120, Thailand

**Keywords:** point-of-care (PoC) testing, MyG6PD, portable diagnostic device

## Abstract

Glucose-6-phosphate dehydrogenase (G6PD) deficiency is a common enzymopathy with significant clinical implications, particularly in malaria-endemic regions and in the management of neonatal hyperbilirubinemia. Timely and accurate detection of G6PD deficiency is critical to prevent life-threatening hemolytic events following oxidative drug administration. This study evaluated the MyG6PD device, a quantitative point-of-care (PoC) tool, for the assessment of hemoglobin concentration and G6PD enzyme activity. Analytical performance was benchmarked against laboratory spectrophotometry and the STANDARD G6PD Analyzer™ (SD Biosensor; Suwon-si, Republic of Korea). MyG6PD demonstrated excellent linearity (R^2^ ≥ 0.99), accuracy (bias < ±15%), and precision (CV < 15%) across normal, intermediate, and deficient activity ranges, including heterozygous females with intermediate phenotypes. The device’s compact, battery-operated design, rapid turnaround, and minimal training requirements support its use in decentralized and resource-limited settings. Furthermore, cost-effective consumables and robust detection of intermediate activity highlight its potential for large-scale deployment. Overall, MyG6PD provides a reliable, accessible, and clinically actionable solution for urgent G6PD deficiency screening, enabling safer administration of oxidative therapies and improving patient outcomes in high-risk populations.

## 1. Introduction

Glucose-6-phosphate dehydrogenase (G6PD) deficiency is the most common enzymatic disorder globally, affecting over 400 million individuals, particularly in malaria-endemic regions of Africa, Southeast Asia, and the Mediterranean [1,2]. This X-linked genetic condition results from more than 185 known mutations in the *G6PD* gene, each leading to variable reductions in enzyme activity [3]. As the rate-limiting enzyme in the pentose phosphate pathway (PPP), G6PD catalyzes the production of nicotinamide adenine dinucleotide phosphate (NADPH), a critical cofactor that protects red blood cells (RBCs) from oxidative stress. Since RBCs lack mitochondria and depend solely on the PPP for NADPH generation, G6PD activity is vital for maintaining cellular redox balance and preventing oxidative damage. A deficiency in G6PD compromises this protective mechanism, making RBCs susceptible to hemolysis in response to certain infections, oxidative drugs (especially antimalarials such as primaquine and tafenoquine), and foods like fava beans [4,5]. This risk is significant in the context of malaria elimination programs, where 8-aminoquinoline-based therapies for radical cure require prior G6PD screening to avoid severe hemolytic reactions [2]. Because the *G6PD* gene is located on the X chromosome, the condition manifests differently across sexes. Hemizygous males typically exhibit either a normal or deficient phenotype, while heterozygous females can present a wide range of enzyme activities due to X-chromosome inactivation (lyonization). This mosaic expression results in intermediate phenotypes, with enzyme activity ranging from approximately 30% to 80% of normal levels [1]. The World Health Organization (WHO) categorizes G6PD activity into three clinical classes: deficient (<30% activity), intermediate (30–80%), and normal (>80%) [6]. Identifying individuals in the intermediate range, particularly heterozygous females, remains a diagnostic challenge but is crucial for safe clinical management. Beyond malaria, G6PD deficiency significantly contributes to neonatal health complications. It is a well-established risk factor for neonatal hyperbilirubinemia (NH) and kernicterus—conditions that, if untreated, may result in irreversible neurological damage or death [7,8]. Notably, both deficient neonates and heterozygous females with intermediate activity are at increased risk [6,9]. Accurate quantification of G6PD activity is therefore essential in clinical and public health contexts—for guiding antimalarial therapy, preventing drug-induced hemolysis, and identifying neonates at risk for severe jaundice. This highlights the need for reliable and accessible G6PD diagnostic tools, particularly in low-resource, high-burden settings.

Currently, quantitative spectrophotometric assays remain the gold standard for measuring G6PD activity due to their high accuracy across the full activity spectrum [2]. However, these assays require specialized laboratory infrastructure, temperature-controlled environments, cold chain logistics, and skilled personnel, which limits their feasibility in decentralized or field-based care [10,11,12]. Various point-of-care (PoC) diagnostics have been developed. Early methods, such as the fluorescent spot test (FST), offered a semi-quantitative assessment but required basic laboratory setups and showed low sensitivity for intermediate phenotypes [10,11,13]. More recent qualitative lateral flow assays, including CareStart™ (Access Bio, Inc., Somerset, NJ, USA) and BinaxNOW™ (Abbott Diagnostics Scarborough, Inc., Scarborough, ME, USA) provide operational simplicity but only detect severe deficiency (<30% activity), failing to identify heterozygous females with intermediate activity [8,14,15]. Quantitative PoC biosensors such as the STANDARD™ G6PD Analyzer have addressed part of this gap by offering enzyme activity normalized to hemoglobin concentration (U/g Hb). However, existing devices still face challenges with intermediate phenotype detection, operational complexity, cost, and scalability for widespread use [12,16,17].

To overcome these limitations, the MyG6PD device was developed as a next-generation quantitative PoC solution combining laboratory-grade analytical reliability with the portability and usability required for field deployment. The device offers several distinct advantages: (i) high analytical performance, with accuracy and precision comparable to spectrophotometry across deficient, intermediate, and normal activity ranges; (ii) robust detection of intermediate phenotypes, addressing a critical gap for safe administration of oxidative drugs in malaria elimination programs; (iii) operational simplicity, requiring minimal training and delivering rapid results at the PoC; and (iv) portability and cost-effectiveness, supported by a compact design, battery operation, and low-cost consumables, making it suitable for deployment in remote, resource-constrained settings. This study evaluates the analytical validity and clinical relevance of MyG6PD through comparison with the reference spectrophotometric method and a widely used commercial PoC device.

## 2. Materials and Methods

### 2.1. Chemicals and Reagents

The following chemicals and reagents were used for the preparation of standard curves, hemoglobin quantification, and glucose-6-phosphate dehydrogenase (G6PD) activity assays. Human hemoglobin standard was provided by Sigma-Aldrich (St. Louis, MO, USA; Cat. No. H7379). Drabkin’s reagent (Cyanmethemoglobin method) and Brij L23 solution were obtained from Sigma-Aldrich (St. Louis, MO, USA; Cat. No. D5941 and B4184) and used for the spectrophotometric determination of hemoglobin at 540 nm. Triton^TM^ X-100 was supplied by Sigma-Aldrich (Cat. No. X100). β-Nicotinamide adenine dinucleotide phosphate, oxidized form (NADP^+^) was provided by Sigma-Aldrich (Cat. No. N0505). D-glucose-6-phosphate disodium salt hydrate (G6P) was supplied by Sigma-Aldrich (Cat. No. G7879). ‘Normal’ (1409 ± 423 U/L), ‘Intermediate’ (451 ± 135 U/L), and ‘Deficient’ (110 ± 72 U/L) G6PD controls were obtained from Pointe G6PD Control Set (Horiba Instruments Inc., Irvine, CA, USA; Cat. No GT583-CT). Distilled or deionized water was used for reagent preparation and dilution.

All chemicals were of analytical grade, and reagents were handled according to the manufacturers’ specifications. Stock solutions of NADP^+^ and G6P were prepared fresh, aliquoted, and stored on ice during assays to prevent degradation and maintain enzymatic stability.

### 2.2. Instrumentation and Measurement Platform of MyG6PD

**Figure 1:**
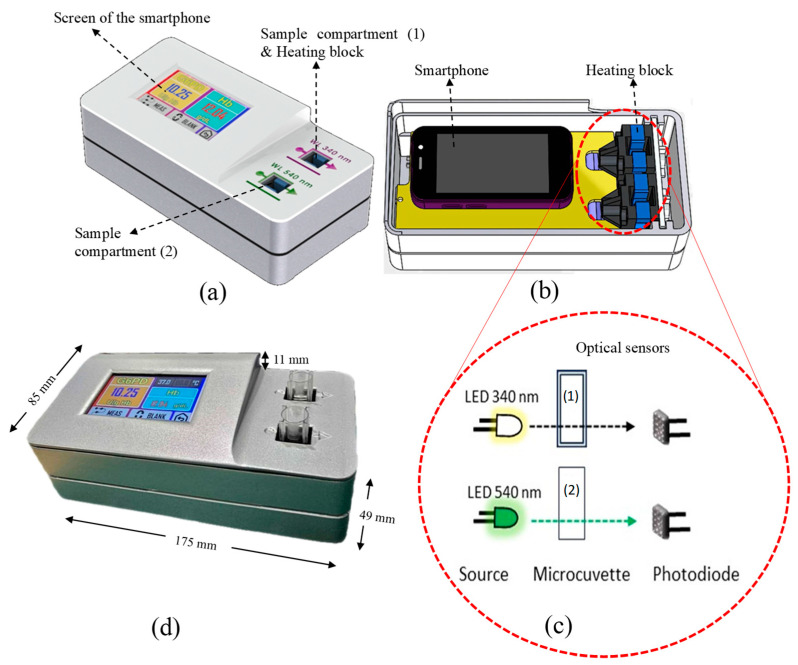
MyG6PD device and measurement platform. (**a**) A portable device with an electronic unit and a smartphone interface for guided operation and data processing. (**b**) Dual-wavelength optical system with 340 nm and 540 nm LEDs and photodiodes for simultaneous measurement of G6PD activity and hemoglobin. (**c**) Disposable UV-transparent microcuvette (15 mm height) maintained at 37 °C by an aluminum block heater for optimal enzymatic reaction. (**d**) Photo image of the device.

**Figure 2:**
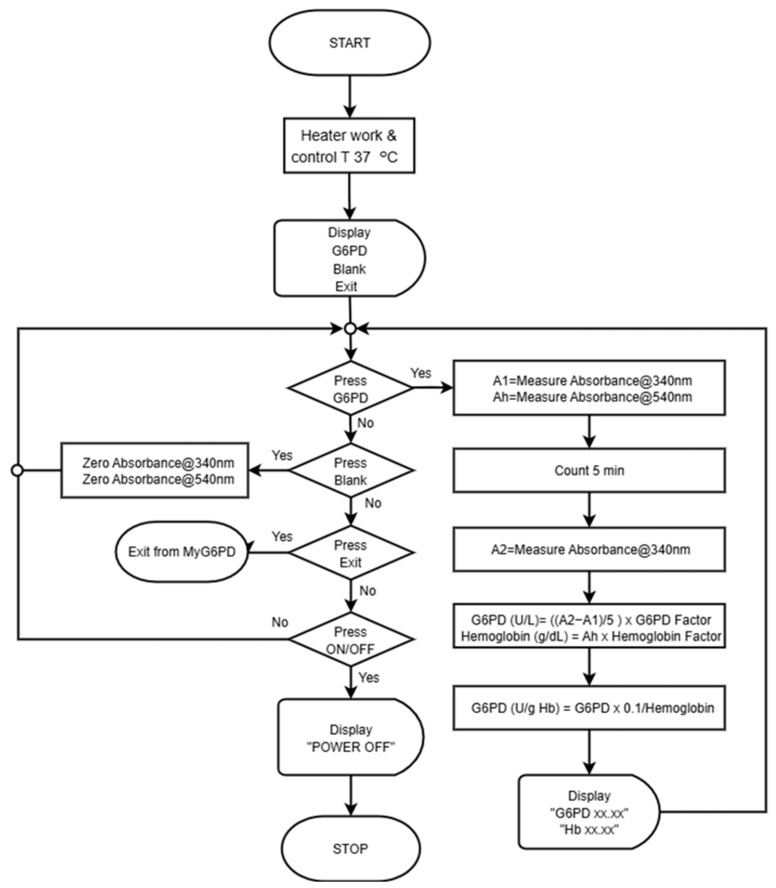
Schematic diagram showing the operational workflow of MyG6PD that controls the MyG6PD application.

MyG6PD is a portable, colorimetric-based device that uses dual optical sensors to simultaneously measure hemoglobin (Hb) levels and G6PD enzyme activity, as illustrated in Figure 1a. The development of the MyG6PD device began with a clear objective, i.e., to create a portable, rapid, and reliable platform capable of simultaneously measuring hemoglobin (Hb) levels and G6PD enzyme activity in a point-of-care setting. To achieve this, the design integrated a dual optical sensing approach, leveraging the distinct absorbance peaks of NADPH and hemoglobin at 340 nm and 540 nm, respectively. This dual-wavelength detection strategy was implemented using two LEDs as light sources and two aligned photodiodes for precise optical signal capture (Figure 1b). A critical aspect of the device was ensuring optimal enzymatic reaction conditions. This issue was addressed by incorporating an aluminum block heater capable of maintaining the microcuvettes at a stable 37 °C, thereby enabling consistent and accurate measurements. To minimize reagent and sample consumption, disposable UV-transparent microcuvettes were employed, designed for simple handling and efficient thermal transfer (Figure 1c). The electronic unit was coupled with a dedicated Android-based smartphone interface (Figure 1a), which guided the user through the assay steps (Figure 2), received optical signals, and processed the data in real time. The software algorithms converted raw absorbance readings into hemoglobin concentration and G6PD activity, normalized per gram of hemoglobin, and displayed results clearly within minutes. Users could store results locally or transmit them remotely for clinical monitoring, ensuring both convenience and traceability. Power management was addressed through an in-house 12 V lithium-ion battery pack, enabling multiple tests in off-grid environments and further enhancing the device’s applicability in diverse clinical and field settings. Throughout the development process, rigorous calibration and validation were conducted. Optical sensors were standardized against reference solutions, and thermal stability was confirmed across microcuvettes. Analytical accuracy was benchmarked against conventional laboratory assays. User testing refined the smartphone interface to ensure intuitive operation and minimal training requirements. Ultimately, the integration of dual-wavelength optical detection, precise thermal control, portable electronics, and a user-friendly smartphone interface yielded a compact and robust device (Figure 1a–c). The photo image of the device is shown in Figure 1d. MyG6PD successfully addresses key limitations of conventional point-of-care and laboratory assays, providing rapid, reliable, and portable screening for both hemoglobin levels and G6PD activity, with potential applications across diverse healthcare and field settings.

### 2.3. Determination of Hemoglobin and G6PD Enzyme Activity

#### 2.3.1. Hemoglobin

Hemoglobin concentration was determined by cyanmethemoglobin method [18]. A stock standard solution of cyanmethemoglobin (210 mg/mL hemoglobin) was prepared using Drabkin’s solution as the diluent. This stock solution was stored at 2–8 °C in a tightly sealed, light-protected container.

Venous blood sample (10 µL) was added to Drabkin’s reagent (2.5 mL). The mixture was left at room temperature (25 °C) for 15 min to ensure complete conversion of hemoglobin to cyanmethemoglobin. The absorbance (at 540 nm) was read against a reagent blank (Drabkin’s reagent without blood) using MyG6PD.

#### 2.3.2. G6PD Activity

The quantitative G6PD activity assay by spectrophotometer is based on measuring the rate of NADPH production catalyzed by the G6PD enzyme in red blood cells (RBCs) [19]. G6PD catalyzes the first step in the pentose phosphate pathway (PPP), converting glucose-6-phosphate (G6P) into 6-phosphogluconolactone, while simultaneously reducing nicotinamide adenine dinucleotide phosphate (NADP^+^) to its reduced form, NADPH.

G6PD activity was measured using a two-reagent system. First, 100 µL of Reagent 1 (R1), containing 1.5 nM NADP and 12 mM maleimide, was dispensed into a microcuvette, followed by the addition of 1 µL of venous blood. The mixture was incubated at room temperature (25 °C) for 10 min to allow pre-reaction equilibration. Subsequently, 200 µL of Reagent 2 (R2), comprising 1.05 mM glucose-6-phosphate (G6P), buffer, and magnesium salts, was added to initiate the enzymatic reaction. The microcuvette was then incubated at 37 °C within the MyG6PD device. Absorbance at 340 nm was measured after 5 min (A_1_) and 10 min (A_2_).

The rate of the reaction (ΔA/min), which reflects the catalytic conversion of NADP^+^ to NADPH by the G6PD enzyme, was calculated using the following formula:

ΔA/min = A_2_ − A_1_/5

where A**_1_** is the initial absorbance at 340 nm, and A**_2_** is the absorbance measured after 10 min. This calculation enables the normalization of G6PD activity to RBC mass, allowing for a direct comparison of enzyme activity between individuals with different hemoglobin levels.

To assess the robustness and thermal sensitivity of the G6PD assay, measurements were conducted on a control sample with a known expected G6PD activity of 280 U/L (the deficiency threshold), across three incubation temperatures: 25 °C (room temperature), 30 °C, and 37 °C (physiological temperature). The results show good stability of G6PD activity up to 37 °C.

The enzymatic activity of G6PD, expressed in units per litre (U/L), was calculated based on the rate of NADPH formation, using the following formula:

$$G6\mathrm{PD}\;(U/L)\;=\;\frac{\Delta A/min\times 1000\times 301\times TCF}{6.22}$$

where ΔA/min is the change in absorbance per minute (rate of reaction) at 340 nm; 301 is the ratio of total reaction and sample volume; 6.22 L/mmol/cm is the millimolar absorptivity of NADPH at 340 nm; and TCF is the temperature correction factor, applied to normalize enzyme activity at the assay temperature (37 °C).

The G6PD enzymatic activity relative to hemoglobin concentration was calculated according to the following equation:

$$G6\mathrm{PD}\;(U/g\;\mathrm{Hb})\;=\;\frac{\Delta A/min\times 100\times 301\times TCF}{6.22\times Hb(g/dL)}$$

where Hb (g/dL) is the hemoglobin concentration in the sample.

### 2.4. MyG6PD Assay Performance

#### 2.4.1. Linearity

The linearity of hemoglobin and G6PD concentrations, as determined by MyG6PD, was established in comparison with those determined by a spectrophotometer (reference method), using the procedures described in Section 2.3. The spectrophotometer used in the study was a UV-visible model (PERSEE; Beijing, China). The size of the microcuvettes used was 12.5 × 12.5 × 45 mm (width × length × height), with an optical path length of 10 mm.

#### Hemoglobin

Linearity of hemoglobin determined by MyG6PD and the reference spectrophotometer was constructed using standard concentrations of 2.4, 4.8, 9.0, 10.3, 14.5, and 20.7 g/dL (triplicate for each concentration).

#### G6PD

Since standard G6PD is not available, control samples at three known levels, i.e., normal (1409 ± 423 U/L), intermediate 451 ± 135 U/L), and deficient (110 ± 72 U/L), were used in the study as stock solutions. Each was further diluted to two activity levels (75% and 90%). Final activity levels (mean value) were 70, 145, 194, 227, 341, 455, 557, 835, and 1113 U/L.

#### 2.4.2. Limit of Quantification

The limit of quantification (LOQ) for hemoglobin and G6PD concentrations was defined as the lowest hemoglobin and G6PD concentrations in control samples that yielded a coefficient of variation (CV) within ±15% of the target concentration.

#### 2.4.3. Precision

The precision of the MyG6PD for detecting hemoglobin and G6PD concentrations in RBCs was evaluated in comparison with the reference spectrophotometer method. The intra-day precision (repeatability) was evaluated using control samples and analyzed on the same day. The inter-day precision (reproducibility) was evaluated using triplicate control samples and analyzed over the three consecutive days. The three concentration levels used for hemoglobin analysis were 2.4, 10.3, and 20.7 g/dL (triplicate each). For G6PD, the three sets of control samples as described above were used (triplicate each) for precision evaluation. Precision was expressed as the coefficient of variation (CV):

%CV = (Standard deviation/Mean) × 100

#### 2.4.4. Accuracy

The accuracy of the MyG6PD in detecting hemoglobin and G6PD concentrations in RBCs was evaluated in comparison with values measured by a spectrophotometer (reference values). Intra-day (repeatability) and inter-day (reproducibility) variability in the assay accuracy of hemoglobin and G6PD were determined using three concentration levels as described above (Section 2.4.3). The intra-day accuracy was evaluated on the same day of analysis, and the inter-day accuracy was evaluated over the three consecutive analysis days.

Accuracy of hemoglobin and G6PD analysis determined by MyG6PD was calculated using the following formula:

Accuracy (%) = [(Mean measured value by MyG6PD − Mean measured value by spectrophotometer)/Mean measured value by spectrophotometer] × 100

### 2.5. Clinical Application of MyG6PD

The clinical application of MyG6PD was evaluated in comparison with the reference spectrophotometer and the commercial STANDARD G6PD Analyzer^TM^ (SD Biosensor; Suwon-si, Republic of Korea), using clinical samples (EDTA venous blood or capillary blood) collected from 15 healthy subjects. In addition, the three levels of control samples were also included in the analysis. The study received ethical approval from the Ethics Committee of the Faculty of Medicine, Thammasat University (Project No. MTU-EC-OO-2-194/66; Approval No. 218/2023), and was conducted at Thammasat Chalermprakiet Hospital and the Thammasat University Center of Excellence in Pharmacology and Molecular Biology of Malaria and Cholangiocarcinoma, in accordance with the principles of the Declaration of Helsinki. Since the project used leftover samples from health check-ups of healthy subjects, consent is not required.

#### 2.5.1. Correlation Analysis

G6PD activity (expressed as U/g hemoglobin) of all 15 blood samples was measured using MyG6PD in comparison with the reference spectrophotometer and the STANDARD G6PD Analyzer^TM^. Correlation analysis of the linear relationship was performed using the Pearson correlation test at a statistical significance level of α = 0.05.

#### 2.5.2. Stability Analysis

Short-term stability of the test for G6PD activity was evaluated by measuring G6PD activity in blood samples collected from all subjects on the same day (day 1) and on days 2 and 5 after collection (storage at 4 °C). G6PD activity (U/g Hb) in all samples, including control samples was determined using MyG6PD, in comparison with the reference spectrophotometer and the STANDARD G6PD Analyzer^TM^. The results are summarized as the mean deviation of the measured values from the values measured on day 1 as follows:

Mean deviation (%) = [(Mean measured value on day 1 − Mean measured value on day 2 or 5)/Mean measured value on day 1] × 100

## 3. Results

### 3.1. MyG6PD Assay Performance

#### 3.1.1. Linearity

##### Hemoglobin

**Figure 3:**
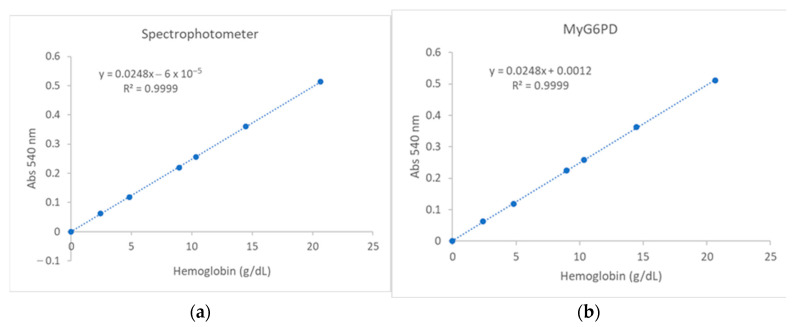
Linearity of hemoglobin determination by (**a**) the reference spectrophotometer and (**b**) MyG6PD.

The linearity (mean from three replicates) for hemoglobin determination by the reference spectrophotometer and MyG6PD demonstrates a strong linear correlation between hemoglobin concentration (g/dL) and absorbance at 540 nm (Figure 3a,b). The regression equation for the reference spectrophotometer was y = 0.0248x − 0.00006. The coefficient of determination (R^2^) of 0.9999 indicates excellent linearity and high precision across the measured range. The regression equation for MyG6PD was y = 0.0248x − 0.0012. The R^2^ of 0.9999 indicates excellent linearity and high precision across the measured range.

##### G6PD

**Figure 4:**
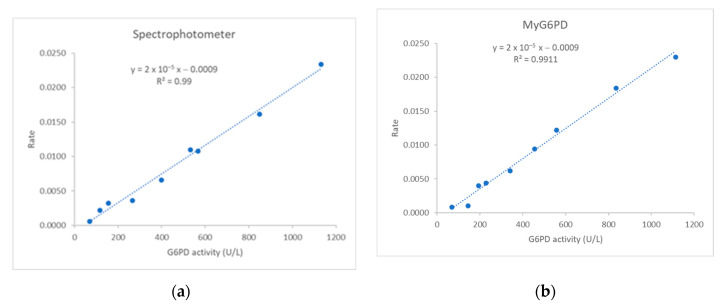
Linearity of G6PD activity determination by (**a**) laboratory spectrophotometer and (**b**) MyG6PD.

The linearity (mean from three replicates) for determining G6PD concentrations by the reference spectrophotometer and MyG6PD demonstrate a strong linear correlation between G6PD activity (U/L) and the rate of enzyme reaction (ΔA/5min) at the concentration range 70 to 1113 U/L (Figure 4a,b). The regression equation for the reference spectrophotometer was y = 0.6324 − 0.0009. The R^2^ of 0.990 indicates excellent linearity and high precision across the measured range. The regression equation for MyG6PD was y = 0.6324 − 0.0009. The R^2^ of 0.9911 indicates excellent linearity and high precision across the measured range.

#### 3.1.2. Limit of Quantification

The LOQ of hemoglobin and G6PD concentrations in the clinical blood samples measured using MyG6PD compared with those measured using the reference spectrophotometer were 2.4 g/dL and 70 U/L, respectively.

#### 3.1.3. Precision and Accuracy

##### Hemoglobin

Precision: Both MyG6PD and the reference spectrophotometer demonstrate excellent precision in determining hemoglobin concentrations. The %CVs of MyG6PD are slightly higher but remain within the acceptable threshold of ≤2–3%, especially for clinical assays (Table 1).

Accuracy: MyG6PD shows good agreement with the reference method, with biases ranging from +2.3% to −1.8%; all are within the acceptable limit of ±15%. The results confirm that MyG6PD provides clinically reliable measurements (Table 1).

##### G6PD

Precision: All %CVs for MyG6PD are ≤15%, which meet general precision standards for diagnostic enzyme assays. Spectrophotometer has slightly better precision, particularly at normal activity levels. MyG6PD performs well, especially at intermediate and deficient levels, which are clinically most important for screening G6PD deficiency (Table 2).

Accuracy: All MyG6PD accuracy values are within ±15%, which meet general diagnostic acceptance criteria. Slight underestimation at normal levels and slight overestimation at deficient levels are not clinically significant. Inter-day accuracy at intermediate level (−12.8%) is at the threshold, suggesting the need for monitoring stability over time (Table 2).

### 3.2. Clinical Application of MyG6PD

#### 3.2.1. Correlation Analysis

**Figure 5:**
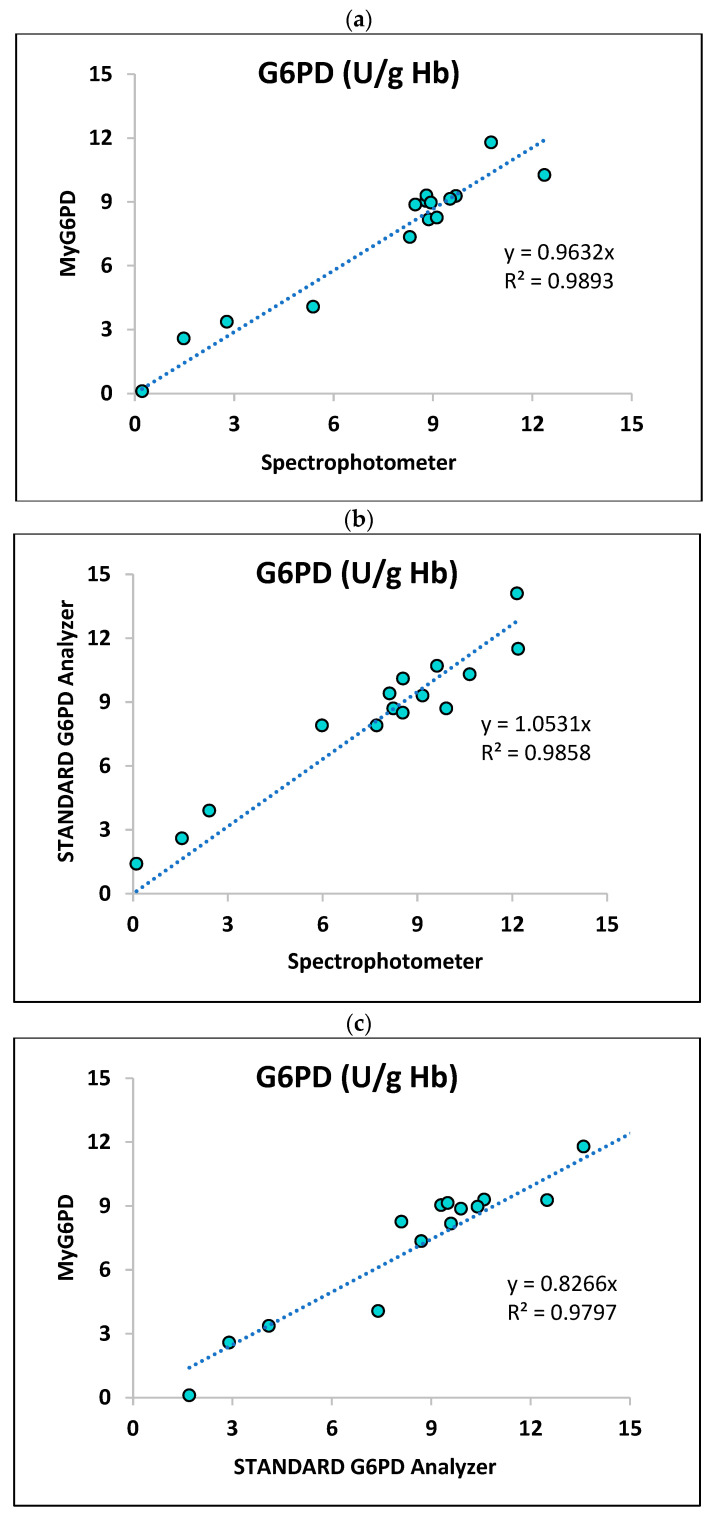
Correlation between G6PD activity (U/g Hb) measured by (**a**) MyG6PD vs. spectrophotometer, (**b**) STANDARD G6PD Analyzer^TM^ vs. spectrophotometer, and (**c**) MyG6PD vs. STANDARD G6PD Analyzer^TM^.

Linear regression analysis shows a strong relationship between G6PD activity (U/g Hb) measured by MyG6PD vs. the reference spectrophotometer (Figure 5a), the STANDARD G6PD Analyzer^TM^ vs. the reference spectrophotometer (Figure 5b), and MyG6PD vs. the STANDARD G6PD Analyzer^TM^ (Figure 5c) across the measured range. The respective regression equations and R^2^ were y = 0.9632x (R^2^ = 0.9313), y = 1.531x (R^2^ = 0.8931), and y = 0.8266x (R^2^ = 0.8695). The results suggest that MyG6PD has high analytical validity in measuring G6PD activity.

#### 3.2.2. Stability Analysis

**Figure 6:**
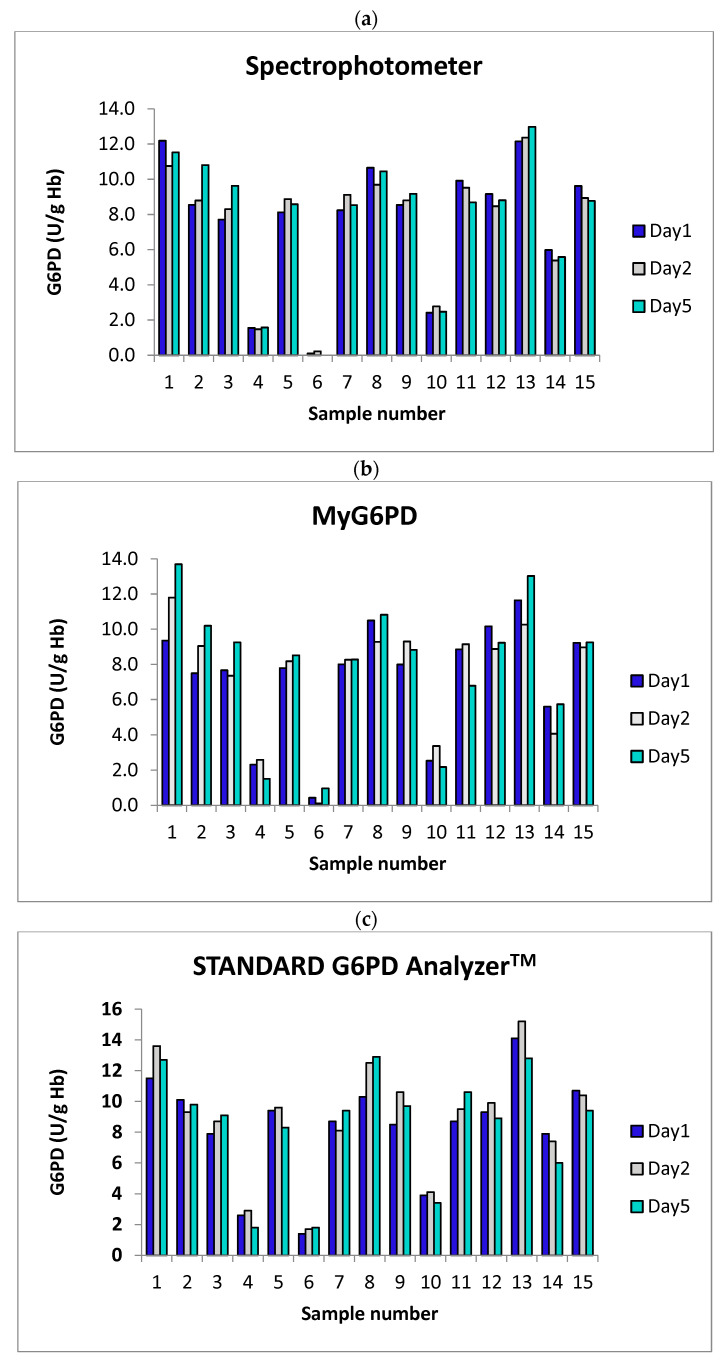
Short-term stability of G6PD activity (U/g Hb) measured by (**a**) the reference spectrophotometer, (**b**) MyG6PD, and (**c**) the STANDARD G6PD Analyzer^TM^. Blood samples were collected from 15 healthy subjects, and the analysis was performed on day 1 (the day of blood collection) and on days 2 and 5 after blood collection (storage at 4 °C).

Blood samples stored at 4 °C for up to 5 days show minor decreases (~10%) in measured G6PD activity across all testing methods (Figure 6, Table 3 and Table 4). The MyG6PD device’s stability performance is comparable to that of the reference spectrophotometer, confirming its reliability for short-term sample storage. These findings underscore the importance of maintaining consistent storage conditions or performing timely testing to ensure accurate G6PD measurement. If more extended storage is expected, further stability studies should be considered.

## 4. Discussion

While significant progress has been made in decentralizing G6PD testing through qualitative and quantitative PoC tools, existing limitations in sensitivity, specificity, and user dependence, especially for intermediate cases, underscore the ongoing need for robust, accurate, and field-appropriate diagnostic solutions. The continued development and validation of novel platforms with improved analytical performance are essential for ensuring equitable and safe access to G6PD-sensitive therapies in global health programs. This study comprehensively evaluated the analytical performance of the MyG6PD device for quantifying hemoglobin concentration and G6PD enzyme activity, benchmarked against a laboratory reference spectrophotometer and the commercial STANDARD G6PD Analyzer^TM^. The findings underscore the device’s potential as a reliable, portable, and accessible tool for PoC G6PD deficiency screening and anemia assessment, particularly in malaria-endemic areas and resource-limited settings. Beyond enabling the simultaneous measurement of G6PD activity and hemoglobin from a single capillary blood sample, it offers improvements in portability, cost-effectiveness, and user-friendliness, thereby reducing dependence on laboratory facilities and trained personnel. Furthermore, its design minimizes sample volume requirements and processing steps, making it particularly advantageous for neonatal screening and large-scale population testing in low-resource settings. These enhancements collectively improve diagnostic efficiency and accessibility, potentially increasing the uptake of G6PD testing and reducing the risk of hemolytic complications in vulnerable populations.

### 4.1. Analytical Performance

Linearity and correlation: MyG6PD demonstrated excellent linearity across clinically relevant ranges, with R^2^ values of ≥0.99 for both hemoglobin and G6PD activity, when compared to the reference spectrophotometer. Additionally, the device showed strong correlation with the SD Biosensor, albeit slightly lower (R^2^ = 0.87), which aligns with reported variabilities among current PoC devices [20].

Precision and reproducibility: Intra-day and inter-day reproducibility assessments confirmed acceptable precision, with CVs consistently under 15% across all activity ranges—normal, intermediate, and deficient. These precision levels meet internationally accepted analytical standards for enzymatic assays. Notably, MyG6PD’s performance at the intermediate activity range, the most challenging for PoC devices, was particularly encouraging and suggests the potential to overcome a key diagnostic gap in identifying heterozygous females at risk for hemolysis.

Accuracy and bias: Accuracy analysis showed deviations of <±15% from reference values across all G6PD activity levels. The regression slope close to unity and minimal intercept bias further confirmed that MyG6PD readings are interchangeable with spectrophotometric results. This level of accuracy is crucial given the role of G6PD activity quantification in guiding the safe use of drugs that induce oxidative stress.

Limit of quantification and sensitivity: The device showed an LOQ of 2.4 g/dL for hemoglobin and 70 U/L for G6PD activity, which is within clinically significant thresholds. This degree of sensitivity is sufficient for the identification of anemic individuals and those with G6PD deficiency, including borderline or partially deficient cases who might otherwise remain undetected with qualitative methods [2].

Short-term stability: The observed stability of G6PD activity in blood samples stored at 4 °C for up to five days is consistent with previously reported enzymatic stability profiles [16]. This result has practical implications for decentralized and resource-limited settings, where immediate testing is often not feasible. The ability to maintain accurate readings under short-term storage conditions supports the use of delayed or batch-mode testing workflows without jeopardizing result integrity. However, if extended storage beyond five days is anticipated, additional studies are necessary to validate long-term stability under varying conditions.

### 4.2. Clinical and Operational Relevance

Current methods for measuring G6PD activity, including spectrophotometric and fluorescent spot tests, have several limitations that hinder their widespread application in clinical and field settings. The gold standard—the spectrophotometric method—requires laboratory infrastructure, skilled personnel, and significant processing time, making it unsuitable for resource-limited environments. Similarly, PoC devices available on the market, while more portable, often face challenges such as variable accuracy under different environmental conditions, the need for venous blood samples, and difficulty in simultaneously assessing hemoglobin levels, which is crucial for accurately interpreting G6PD activity in relation to anemia status. MyG6PD’s quantitative output, rapid turnaround time, compact design, and minimal training requirements make it a strong candidate for routine screening in primary care clinics, rural health posts, and mobile diagnostic units. Both MyG6PD and the STANDARD G6PD Analyzer^TM^ provide an alternative platform for measuring G6PD activity. Functionally, systems provide G6PD activity normalized to hemoglobin (U/g Hb), their biochemical readouts and detection mechanisms differ significantly. While both MyG6PD and the STANDARD G6PD Analyzer^TM^ offer valuable platforms for quantitative G6PD testing, they operate on fundamentally different principles. MyG6PD relies on spectrophotometer-based analysis, whereas the STANDARD G6PD Analyzer^TM^ uses reflectance-based colorimetry. In contrast to existing PoC tools such as CareStart™, which provide only qualitative results with limited sensitivity for intermediate phenotypes. MyG6PD delivers robust numerical values that support more nuanced clinical decisions [8]. Importantly, accurate identification of individuals with intermediate G6PD activity, especially heterozygous females, who traditional methods may not reliably detect is a critical advancement enabled by MyG6PD. This allows more personalized therapeutic risk assessment in malaria elimination programs and neonatal hyperbilirubinemia prevention efforts.

Compared to laboratory spectrophotometry, MyG6PD offers significant advantages in portability, ease of use, and operational feasibility in resource-limited settings. The device features a compact, battery-operated design, achieves rapid turnaround times, and requires minimal operational training. These features support deployment in primary healthcare facilities, mobile units, and malaria-endemic areas where timely G6PD assessment is critical. When compared to the STANDARD G6PD Analyzer™, MyG6PD provides comparable overall accuracy but exhibits a distinct advantage in detecting intermediate G6PD activity, which is clinically important for identifying heterozygous females at risk of hemolysis. Intermediate classification remains a major limitation of qualitative and some quantitative PoC tools; however, MyG6PD demonstrated superior reproducibility and lower variability in this range (CV < 15%) compared with the STANDARD G6PD Analyzer™, which ensures reliable differentiation between intermediate and normal phenotypes. Additionally, MyG6PD’s lower cost per test and simplified maintenance requirements enhance its sustainability for large-scale implementation compared to both spectrophotometric systems and existing commercial PoC analyzers. Despite the advantageous features of MyG6PD, further large-scale validation studies across diverse demographic and geographic populations are recommended. Future studies should also compare its performance against gold-standard methods across varying hematocrit levels and in populations with different G6PD variants to confirm its robustness and reliability. Additional work could also examine the integration of MyG6PD into digital health platforms, its cost-effectiveness, and the feasibility of training for frontline health workers.

## 5. Conclusions

The MyG6PD device demonstrates excellent analytical performance for the simultaneous quantification of hemoglobin concentration and G6PD enzyme activity, with high agreement with gold-standard laboratory spectrophotometry. Based on its precision, accuracy, and operational suitability, MyG6PD represents a valuable tool to advance equitable access to safe G6PD-sensitive therapies and timely diagnosis of hemolytic risk in resource-limited and field settings. MyG6PD improves operational simplicity; further validation under diverse clinical and environmental conditions is necessary to ensure consistent accuracy. Additionally, integration with digital reporting and connectivity could strengthen its role in screening programs.

## 6. Patents

The innovation patent ‘MyG6PD’ is approved by the Department of Intellectual Property of Thailand (No. 250-3002-914).

## Figures and Tables

**Table 1:** Precision and accuracy (intra- and inter-day) for the determination of hemoglobin concentrations in blood using MyG6PD compared with the reference spectrophotometer.

	Hemoglobin Concentration (g/L)	Precision (%CV)		Accuracy (%)	
		Intra-Day	Inter-Day	Intra-Day	Inter-Day
MyG6PD	2.4	0.9	1.2	+2.0	+2.3
	10.3	1.2	1.7	+1.2	+0.9
	20.7	1.8	2.1	−0.19	−1.8
Spectrophotometer	2.4	0.7	1.1	NA	NA
	10.3	0.9	0.9	NA	NA
	20.7	1.2	1.0	NA	NA

NA = Not applicable.

**Table 2:** Precision and accuracy (intra- and inter-day) for the determination of G6PD activity in blood using MyG6PD compared with the reference spectrophotometer.

	G6PD Concentration (U/L)	Precision (%CV)		Accuracy (%)	
		Intra-Day	Inter-Day	Intra-Day	Inter-Day
MyG6PD	1409 ± 423	9.03	12.8	−9.68	−9.4
	451 ± 135	9.95	7.2	+11.67	−12.8
	110 ± 72	14.29	9.8	+11.56	+13.3
Spectrophotometer	1409 ± 423	5.54	10.7	NA	NA
	451 ± 135	8.68	7.6	NA	NA
	110 ± 72	11.17	7.8	NA	NA

NA = Not applicable.

**Table 3:** Stability of G6PD activity (U/g Hb) determined by MyG6PD in comparison with the reference spectrophotometer and the STANDARD G6PD Analyzer^TM^ following storage of blood samples at 4 °C (days 2 and 5) before analysis. Results are expressed as % mean deviation (+SD) from the activity measured on day 1.

**% Mean deviation from Day 1 (SD) value**	**Spectrophotometer**		**MyG6PD**		**STANDARD G6PD** **Analyzer^TM^**	
	Day 2	Day 5	Day 2	Day 5	Day 2	Day 5
	9.7 ± 1.3	10.0 ± 1.9	10.3 ± 2.0	11.4 ± 3.1	11.9 ± 2.3	10.9 ± 2.3

**Table 4:** G6PD activity (U/g Hb) in the three levels of control samples (mean values of triplicate analysis) measured on days 1, 2, and 5 by MyG6PD in comparison with the reference spectrophotometer and the STANDARD G6PD Analyzer^TM^.

	G6PD Activity (U/g Hb)	Day 1	Day 2	Day 5
MyG6PD	11.8 ± 3.5	12.5	8.7	10.9
	4.0 ± 1.2	3.2	3.9	4.6
	0.9 ± 0.6	2.2	1.1	1.9
STNADARD G6PD Analyzer^TM^	11.8 ± 3.5	8.5	12.5	8.3
	4.0 ± 1.2	2.0	2.5	2.0
	0.9 ± 0.6	1.3	1.4	0.9
Spectrophotometer	11.8 ± 3.5	15.1	9.5	10.8
	4.0 ± 1.2	4.9	3.6	3.2
	0.9 ± 0.6	1.0	1.3	0.9

## Data Availability

Data are contained within the article.

## Abbreviations

FST	Fluorescent spot test
g/dL	Grams per deciliter
G6P	D-glucose-6-phosphate disodium salt hydrate
G6PD	Glucose-6-phosphate dehydrogenase
Hb	Hemoglobin
L	Liter
LOQ	Limit of quantification
mM	Millimolar
mmoL	Millimoles
NADP^+^	β-Nicotinamide adenine dinucleotide phosphate
NADPH	Nicotinamide adenine dinucleotide phosphate
NH	Neonatal hyperbilirubinemia
nM	Nanometer
°C	Degrees Celsius
PADs	Paper-based analytical devices
pH	Potential of hydrogen
POC	Point-of-care
PPP	Pentose phosphate pathway
RBCs	Red blood cells
TCF	Temperature correction factor
Tris-HCl	Tris (hydroxymethyl) aminomethane hydrochloride
U/L	Units per litre
vs	Versus
WHO	World Health Organization
